# Juvenile Ovine Ex Vivo Larynges: Phonatory, Histologic, and Micro CT Based Anatomic Analyses

**DOI:** 10.1155/2019/6932047

**Published:** 2019-03-04

**Authors:** Michael Döllinger, Olaf Wendler, Claus Gerstenberger, Tanja Grossmann, Marion Semmler, Hossein Sadeghi, Markus Gugatschka

**Affiliations:** ^1^Division for Phoniatrics and Pediatric Audiology, Department of Otorhinolaryngology, Head and Neck Surgery, Medical School, University Hospital, FAU Erlangen-Nürnberg, Waldstrasse 1, 91054 Erlangen, Germany; ^2^Laboratory for Molecular Biology, Department of Otorhinolaryngology, Head and Neck Surgery, Medical School, University Hospital, FAU Erlangen-Nürnberg, Waldstrasse 1, 91054 Erlangen, Germany; ^3^Division of Phoniatrics, ENT University Hospital Graz, Medical University Graz, Auenbruggerplatz 26, Graz 8036, Austria

## Abstract

It is well known that the phonatory process changes during the life span. However, detailed investigations on potential factors concerned are rare. To deal with this issue, we performed extended biomechanical, macro anatomical, and histological analyses of the contributing laryngeal structures in ex vivo juvenile sheep models. Altogether twelve juvenile sheep larynges were analyzed within the phonatory experiments. Three different elongation levels and 16 different flow levels were applied to achieve a large variety of phonatory conditions. Vocal fold dynamics and acoustical and subglottal signals could be analyzed for 431 experimental runs. Subsequently, for six juvenile larynges microcomputed tomography following virtual 3D reconstruction was performed. The remaining six juvenile larynges as well as six ex vivo larynges from old sheep were histologically and immunohistologically analyzed. Results for juveniles showed more consistent dynamical behavior compared to old sheep larynges due to vocal fold tissue alterations during the life span. The phonatory process in juvenile sheep seems to be more effective going along with a greater dynamic range. These findings are supported by the histologically detected higher amounts of elastin and hyaluronic acid in the lamina propria of the juvenile sheep. The 3D reconstructions of the thyro-arytenoid muscles (TAM) showed a symmetrical shape. Intraindividual volume and surface differences of the TAM were small and comparable to those of aged sheep. However, TAM dimensions were statistically significant smaller for juvenile larynges. Finally, topographical landmarks were introduced for later comparison with other individuals and species. This work resulted in detailed functional, immunohistological, and anatomical information that was not yet reported. This data will also provide reference information for therapeutic strategies regarding aging effects, e.g. laryngeal muscle treatment by functional electrical stimulation.

## 1. Introduction

In vivo investigation of the larynx and especially the vocal folds (VF) is limited due to their small size, their inaccessibility in the in vivo state, and the sensitivity of the microarchitecture. Likewise, image-based investigation is constricted by the limited accessibility [1]. Defining exact laryngeal boundary conditions for phonation, such as adduction and elongation of the VF, is not possible [2]. Biopsies of the laryngeal structures (mucosa, muscles) carry the risk of long-lasting damage of these, going along with a hoarse and breathy voice (dysphonia). As a consequence, most phonatory investigations in humans are restricted to acoustic analysis (perception and quantitative analysis), electroglottagraphy (EGG), and visualization techniques imaging the superior VF surfaces [3, 4]. Also, the complex microarchitecture of the VF (consisting of multi-layered epithelium and the layered lamina propria) as well as the underlying muscle structures can hardly be visualized [5, 6] and not quantified. However, the muscular structures play a crucial role in coordinating the VF dynamics. These muscles are small and their courses lie in different geometrical planes, making it difficult to gather suitable in vivo MRI and CT scans.

Future therapeutic laryngeal treatment options will have to focus more on causal rather than symptomatic treatment, requiring new methods of evaluation and documentation. The consequences of these target-orientated interventions need first to be established in animal models by analysis of morphological and functional (biomechanical) changes before going into clinical trials. For example, a recent study by our group employed an aged sheep model (~ 9 years of age) and demonstrated that, by functional electrical stimulation (FES) of the recurrent laryngeal nerves, a significant change of aged laryngeal muscle dimensions could be achieved [7]. Our studies support previous work that the ovine larynx model is suitable due to its similar size when compared to humans and similar histological features [8].

To enhance our knowledge on biomechanical, macro anatomical, and histological characteristics of the contributing/relevant laryngeal structures and to achieve more insight into aged and juvenile larynges, this study is investigating the following objectives.

1. Analyze the difference between juvenile and aged ovine larynges in dynamics, anatomy, and histology.
2. Identify and quantify dependencies between applied subglottal pressure and elongation towards aero-acoustic parameters in juvenile sheep. Compare the results to previous findings for old/aged sheep larynges [9].
3. A wide range of phonatory conditions was applied to get an overview on the phonatory range of juvenile ovine larynges.

This work is a follow-up study of our groups where anatomical and phonatory characteristics of larynges from aged sheep were in focus [7, 9]. In both studies, phonatory experiments and 3D CT reconstruction were performed similarly to ensure comparability of results. Additionally, immunohistological analysis was performed for both juvenile and aged sheep larynges.

## 2. Materials and Methods

### 2.1. Tissue Harvest and Sample Processing

As animal model, we employed twelve juvenile female sheep (i.e., lambs) (merino mountain breed) with an age of less than one year. The larynges were gathered from the local abattoir. The larynges were immediately removed, dissected, and quick-frozen in liquid nitrogen. Subsequently, the specimens were stored in a freezer at -80°C. This ensured that, after thawing, the viscoelastic tissue characteristics of the larynges remained similar to freshly excised larynges [10].

### 2.2. Phonatory Experiments

**Figure 1:**
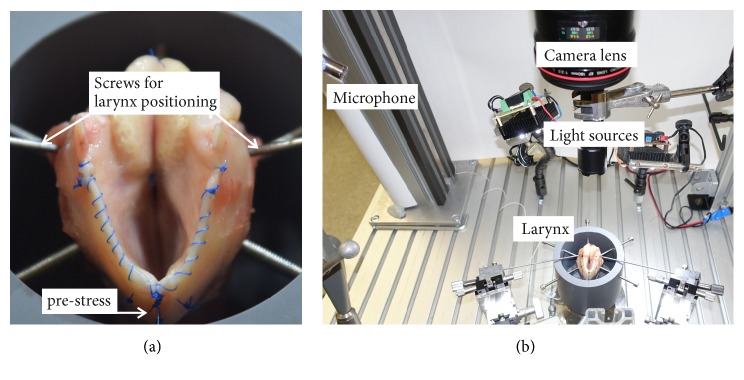
Prepared and fixated sheep larynx (a). The entire setup is given (b); same setup as used in [9] for ex vivo aged sheep larynges.

The experiments were performed* ex vivo* with juvenile cadaver sheep larynges, similar to [9] where the experiments were described in detail. Briefly, the day before the experiments, the larynges were slowly thawed overnight in a refrigerator. The larynges were dissected to expose the VF (Figure 1). The larynges were mounted on an artificial trachea with a diameter of 16 mm, including a drilling for the subglottal pressure sensor 130 mm below the glottis. A custom-made PVC tube prevented unintentional displacement of the larynx. Several screws held the larynx in position.

Three different weights (w_1_ = 20 g, w_2_ = 40 g, w_3_ = 60 g) were attached to the thyroid cartilage to induce pre-stress forces towards the TAM (by tilting the thyroid cartilage against the cricoid cartilage) and to simulate longitudinal tension of the VF (TAM). After the weights were mounted, two iron rods symmetrically adducted the posterior part of the larynx to the point of nearly complete glottis closure, i.e., approximation of the vocal processes to bring the VF in phonatory position [11]. For each larynx, up to 16 runs per pre-stress level w_i_ were performed. By slowly increasing the airflow, the phonation onset level was detected. From there, the applied airflow was successively increased in steps of 2.5 l/min or 5.0 l/min. The experiments were stopped when the larynx did not vibrate any longer (L3 did not vibrate for the last run at w_2_). Three larynges could not be excited to vibration (L1, L2, L5). This yielded altogether 431 runs for the remaining nine larynges for further analysis. For each run, 1.0 s of sustained oscillation was analyzed. All experiments were performed with left-right symmetric pre-stress force.

The dynamics were recorded with a high-speed camera Phantom V2511 (Vision Research Inc., NJ, USA) (4000 fps, 768 x 768 pixels). The experiment was illuminated with a high-power LED flashlight (TK15, Fenix GmbH, Germany). The acoustical signal was recorded 30 cm above the larynx at an angle of 45° at 96 kHz with a 4189 Brüel & Kjaer 1/2-inch free-field microphone (Brüel & Kjaer, Bremen, Germany). The subglottal pressure was measured with a XCS-93-5PSISG Kulite pressure sensor at 96 kHz (Kulite Semiconductor Products, Inc., NJ, USA). Air flow was controlled by a MKS 4000B digital power supply, driving a 1579A/B mass flow controller. The applied air was humidified (ConchaTherm Neptune, Teleflex, Morrisville, North Carolina, USA) and heated (37°C). Signal synchronization and recordings were computer-controlled using the software LabView (National Instruments, TX, USA) [12].

Seven parameters were investigated. Four aerodynamic parameters were computed: airflow and averaged subglottal pressure (P_S_) level. The Cepstral Peak Prominence CPP_S_ (dB) according to Hillenbrand [13, 14], reflecting the periodicity of the time resolved subglottal pressure signal, was computed. The laryngeal flow resistance R_B_ was determined using the definition of van den Berg [15].

Three parameters were computed from the acoustical signal: the sound pressure level SPL (dB), the phonatory fundamental frequency f_0_ (Hz), and again the Cepstral Peak Prominence CPP_A_ (dB) [14].

### 2.3. Micro-Computed Tomography (micro-CT) and 3D Reconstruction

**Figure 2:**
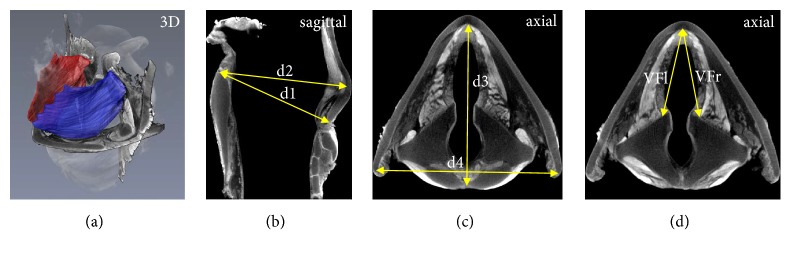
(a) Volume rendering of a sheep larynx with 3D reconstruction of left (blue) and right (red) TAM. Subfigures (b)–(d) show the measured anatomical landmarks and morphometric parameters in the micro-CT scans.

Preparation and contrast agent enhancement were performed as described in detail in our previous study [9]. Briefly*, s*ix larynges were randomly chosen for the 3D reconstruction procedure. After completion of phonatory experiments, the larynges were immersed in 4% phosphate-buffered formalin solution (PBFS) for a period of two days for fixation and then stored for ten days in 3.75 % iodine potassium iodide (I_2_KI) solution as contrast agent to enable the visualization of intrinsic laryngeal muscle structures in the micro-CT scans [16]. A Siemens Inveon micro-CT scanner (Siemens Healthcare GmbH, Erlangen, Germany) was employed to generate high-resolution CT images using a special scan protocol to optimize the visualization of muscle tissue. The resulting 3D image datasets consisted of 1024 slices with an image resolution of 1216 x 1216 pixels and a pixel size of 52 x 52 *µ*m. Image segmentation, 3D reconstruction, and volume/surface determination of the thyro-arytenoid muscle (TAM) were performed with the 3D visualization and analysis software Avizo 9.5 (FEI, Oregon, USA). In order to perform reliable and reproducible measurements when using 3D image datasets, we introduced a number of topographical landmarks and defined distances between them (Figure 2). These are later referred to as morphometric data. Mann-Whitney U tests were applied to investigate differences between old [9] and juvenile sheep larynges.

### 2.4. Histological and Immunohistological Analysis

After the ex vivo experiments, six juvenile and six old larynges (the latter were larynges from the previous study [9]) were histologically and immunohistologically analyzed. All larynges were fixed in 10% buffered formalin. After fixation, the tissues were embedded into paraffin using standard procedures. Each VF was sectioned in longitudinal direction in the middle of the vibrating segment. Successive 6 *µ*m thick sections of each laryngeal specimen were prepared and stained with both van Gieson for collagen distribution and Gomori for reticular fiber distribution [17]. Van Gieson staining was performed in accordance with the manufacturer's instructions (Roth, Karlsruhe, Germany).

Immunostaining on paraffin embedded sections was carried out with ImmPRESS™–AP Anti-Rabbit IgG or Anti-Mouse IgG (alkaline phosphatase) Polymer Detection Kit (Vector Laboratories, Inc., Burlingame, USA). To make the hyaluronic acid-epitopes available for antibody binding the sections underwent deparaffinization and heat-mediated antigen retrieval, using Vector Antigen Unmasking Solution pH 6 (Vector Laboratories, Inc., Burlingame, USA) at 95°C for 25 minutes. For the staining against elastin a proteinase K digestion (0.2 mg/ml proteinase K in 50 mM Tris ph 7.5 for 10 min) was performed.

**Figure 3:**
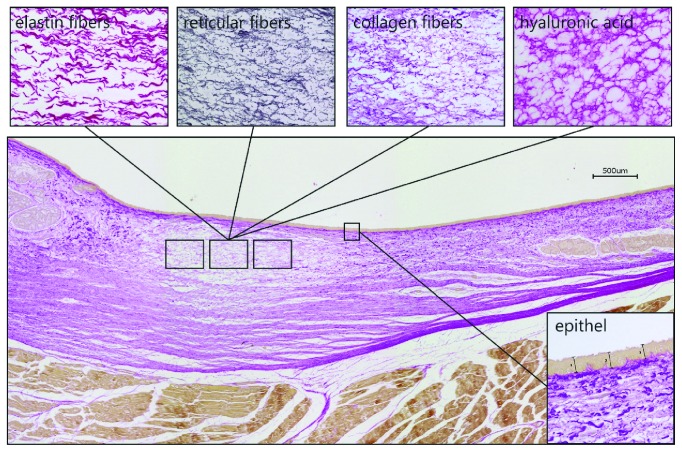
Overview of the connective tissue stainings and the localization of the measurement windows on the exemplary longitudinal section (van Gieson staining) of a juvenile ovine vocal fold.

To avoid unspecific background staining, the slides were incubated for 10 minutes with BLOXALL™ endogenous peroxidase and alkaline phosphatase blocking solution (Vector Laboratories, Inc., Burlingame, USA) and for another 30 minutes with an unspecific protein block (2% horse serum). We used, for elastin staining, the mouse monoclonal antibody clone BA-4 (Santa Cruz Biotechnology, Heidelberg, Germany) and for hyaluronic acid staining a polyclonal affinity purified rabbit antibody (Cloud-Clone Corp., Houston, USA). After incubation with the ImmPRESS™-AP reagent, the slides were developed with chromogen VECTOR-Red Alkaline Phosphatase substrate (Vector Laboratories, Inc., Burlingame, USA). The stained sections were examined with a digital BZ-9000 microscope (Keyence, Neu-Isenburg, Germany) with the software BZ-II-Analyzer and analyzed using ImageJ (version 1.49, National Institutes of Health, Bethesda, MD). For semiquantitative analysis, the staining density of collagen, elastin, hyaluronic acid, and reticular fibers from three representative regions (size: 350 *μ*m × 250 *μ*m ) within the lamina propria (Figure 3) was assessed through densitometry. The thickness of the epithelium was measured as the average over the three measurements. Figure 3 shows a representative longitudinal section through an entire juvenile VF fold to illustrate the different staining and the localization of the measurement windows in the middle of the lamina propria within the main oscillation zone (aka. pars membrancea). Due to the small sample size (n=18 for each group), Mann-Whitney U tests were applied to investigate differences between old and juvenile sheep larynges.

## 3. Results and Discussion

### 3.1. Analysis of Phonatory Experiments

**Figure 4:**
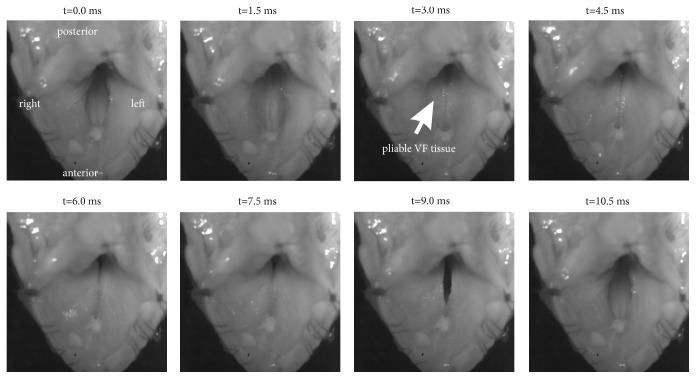
Still images from a high-speed video (L6, w_1_=20g, f_0_=96 Hz) for one oscillation cycle.

The juvenile sheep larynges produced large vibrations and exhibited very soft and pliable VF tissue characteristics (Figure 4) as also observed for the old sheep larynges [9]. Mucosal wave propagation along the VF tissue was distinct but, as seen for old sheep (G), occluded the trachea and glottis (Figure 4). Therefore, the time where the glottis was visibly open (t = 9.0 ms) was very short preventing the accurate segmentation of the glottal area waveform [9]. In summary, 431 recordings for nine larynges were analyzed in the phonatory experiments.

An overview on the parameters' mean values separated for larynges and pre-stress levels is given in Table 1. For phonation onset pressure (PTP), no statistically significant differences (Friedman test, p=0.368) were detected regarding increased pre-stress levels as seen for aged sheep [9].

**Figure 5:**
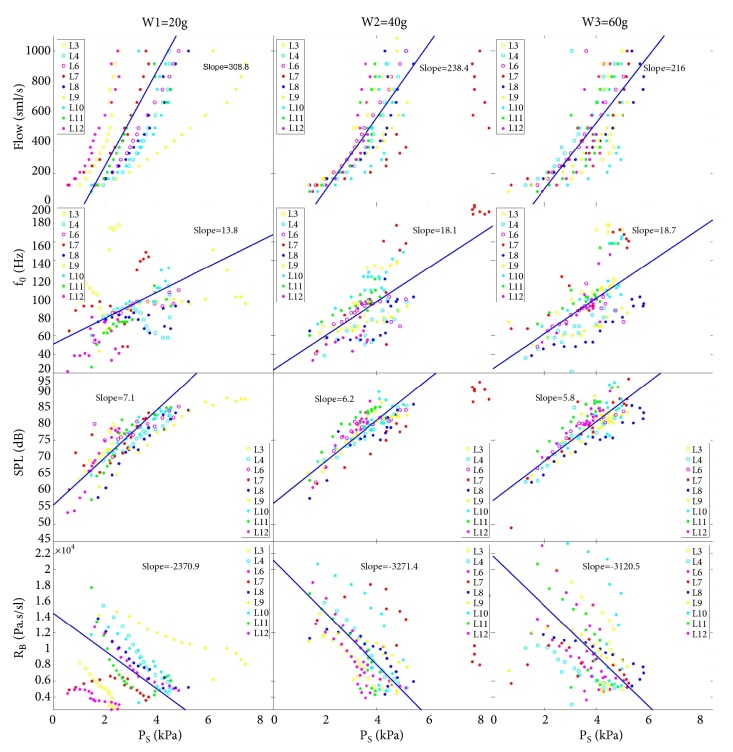
Aerodynamic parameters of the three elongation levels plotted over the subglottal pressure P_S_.

Further, the investigated phonatory parameters were analyzed as functions of subglottal pressure P_S_ and separated for the three pre-stress levels w_i_ (Figure 5). The plotted regression lines are the averaged regressions over the individual larynges.

*P*_*S*_ *vs airflow*: For all three pre-stress levels, the flow was in the same range. However, for low (w_1_, larynx L9) and medium (w_2_, larynx L7) elongation levels, P_S_ reached values of 8000 Pa and above, whereas for the high elongation level P_S_ topped out at 6000 Pa. As found for aged sheep [9], the slope of the regression lines continuously decreased for increasing pre-stress levels; i.e., an increased pre-stress seems to reduce the amount of air necessary to achieve a certain subglottal pressure. The overall range of measured P_S_ (556 Pa–8348 Pa) was higher whereas the applied flow was in a smaller range and lower (83 ml/s–1083 ml/s) than that observed for aged bovines (75 ml/s–1583 ml/s) [9].

*P*_*S*_ *vs f*_*0*_: For all three pre-stress levels, the fundamental frequencies were approximately in the same range (22 Hz–180 Hz); the only exception was larynx L7 at w_2_ that reached up to 200 Hz for the highest P_S_ values. These frequencies were much higher than those found for aged sheep (up to 158 Hz) [9] and in other studies (191 Hz [18]). The slope of the regression lines steadily increased for increasing pre-stress levels from 13.8 Hz/kPa to 18.7 Hz/kPa. This suggests that, for increased pre-stress levels, less pressure increase is needed to achieve higher fundamental frequencies. In contrast, for old sheep, not such tendency was observed [9]. However, the found slopes were in the range or slightly higher than previously reported [9, 19].

*P*_*S*_ *vs SPL*: The slope of the regression lines decreased for increasing pre-stress levels from 7.1 dB/kPa to 5.8 dB/kPA. Again, not such tendency was observed for aged sheep larynges but values were in the same range [9]. Absolute SPL values seemed to slightly increase for higher pre-stress levels.

*P*_*S*_ *vs R*_*B*_: The overall values increased for increasing pre-stress levels. The slopes of the regression lines clearly decreased for the two larger pre-stress levels. The slopes for juvenile sheep were found to be 4 to 8 times greater compared to aged sheep larynges [9]. Also, the overall absolute R_B_ values were on average more than twice as high as those for old sheep, reflecting a higher and improved energy transfer [20] from the glottal flow to the VF in the juvenile larynges. Similar high R_B_ values were previously reported only for rabbit* ex vivo* larynges [21].

**Figure 6:**
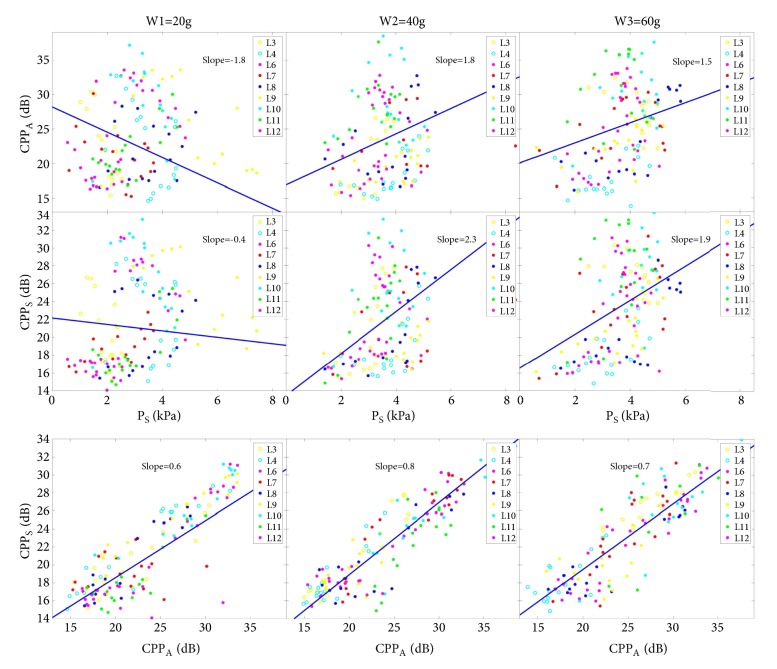
Upper two rows: CPP_A_ and CPP_S_ for the three elongation levels plotted over subglottal pressure P_S_. Lower row: CPP_S_ plotted over CPP_A_.

The CPP was computed for the acoustic and subglottal pressure signal (Figure 6).

*P*_*S*_ *vs CPP*_*A*_: The CPP_A_ values are scattered between 13.9 dB and 38.5 dB over all P_S_ levels; no obvious relation within the individual larynges could be observed. The CPP_A_ slopes of the regression lines did not show a unique tendency for increasing pre-stress. However, as seen for aged sheep larynges, the slope was first negative for the lowest pre-stress level and switched then to positive values [9]. Absolute values were in the same range.

*P*_*S*_ *vs CPP*_*S*_: The CPP_S_ values are scattered between 14.1 dB and 34.7 dB over all P_S_ levels. However, for the two higher pre-stress levels, the correlation to P_S_ seemed to improve. No obvious trend for individual larynges was observed. The CPP_S_ quantities and slopes of the regression lines did not show a unique tendency for increasing pre-stress but behaved similar to CPP_A_.

*CPP*_*A*_ *vs CPP*_*S*_: A clear correlation is observable that did not significantly change over pre-stress levels. CPP_S_ values were found a little smaller compared to CPP_A_ values; i.e., slope < 1. The strong dependency between both CPPs confirms a previous ex vivo rabbit study [21]: There is a direct and strong relation between the occurrence of harmonics in the exciting subglottal flow signal and the development of harmonics in the resulting acoustic signal being emitted by the oscillating VF. However, the CPP values in the current study could not be related to VF dynamics (i.e., glottal closure insufficiency) since the glottal area could not be segmented.

A major shortcoming for the phonatory experiments is that the glottal area could not be segmented and hence the glottal dynamics could not be objectively analyzed and included in the analysis [22]. Hence, correlating aerodynamic parameters towards glottal dynamics was not possible as reported in previous studies on different other species [20, 21, 23].

### 3.2. Micro-CT, Image Segmentation, and 3D Reconstruction

The 3D reconstructions of the TAM yielded symmetrical shapes, and the average percentage volume difference between left and right TAM was in juveniles 2.08 ± 1.01% (Figure 2(a)), which was slightly larger than in aged sheep, where in our previous study a difference of only 1.55 ± 0.85% was determined [9]. The small intraindividual volume differences of the TAM allow assessment and quantification of a unilateral intervention, for example, where changes of a volume need to be proven. Results of volumetric measurement, surface areas, and morphometric data are shown in Table 2. The mean of the lambs TAM volume (left and right combined) measured in this study was 535 ± 86 mm^3^ which is about half the volume of adult sheep (1202 ± 380 mm^3^). The surface areas of the TAM had a similar ratio. Lambs have a mean TAM surface area of 653 ± 85 mm^2^, adult sheep 1100 ± 266 mm^2^. As expected, the morphometric measurements of lambs also showed smaller dimensions. Distance d1, defined as the distance between posterior cranial edge of the cricoid cartilage and inferior thyroid margin, was determined with 31.35 ± 1.91 mm (aged sheep: 36.81 ± 4.10 mm) (Figure 2(b)). The longitudinal distance between posterior cranial edge of the cricoid cartilage and the laryngeal tubercle of the thyroid cartilage (d2) was 32.83 ± 1.68 mm (aged sheep: 41.11 ± 4.10 mm) (Figure 2(b)). The longitudinal distance between posterior cranial edge of the cricoid cartilage and thyroid cartilage in the axial plane (d3) was 31.42 ± 1.52 mm (aged sheep: 40.01 ± 3.74 mm) (Figure 2(c)). Distance d4, defined as the left to right distance of posterior edges of the laminae of the thyroid cartilage at the height of the superior margin of the cricoid cartilage, is measured with 33.98 ± 2.96 mm (aged sheep: 36.21 ± 3.72 mm) (Figure 2(c)). VF lengths, defined as the distance from TAM insertion at vocal process to anterior commissure, were calculated bilaterally, whereas the length of the left VF (VFl) was 17.56 ± 0.70 mm (aged sheep: 22.89 ± 2.43 mm) and the right VF (VFr) was 17.49 ± 0.37 mm (aged sheep: 23.03 ± 2.59 mm) (Figure 2(d)). Juveniles show higher fundamental frequencies (91 ± 33 Hz) compared to aged sheep (83 ± 16 Hz) due to the shorter VF lengths.

Aged [9] and juvenile larynges showed statistically significant differences (p<0.0001) for volume, surface, surface-area-to-volume ratio, and length (VFl and VFr). Also, for the other morphometric data statistically significant differences were found (d1 with p=0.025; d2 and d3 with p= 0.04) except for d4 (p=0.262).

### 3.3. Histological Results

**Figure 7:**
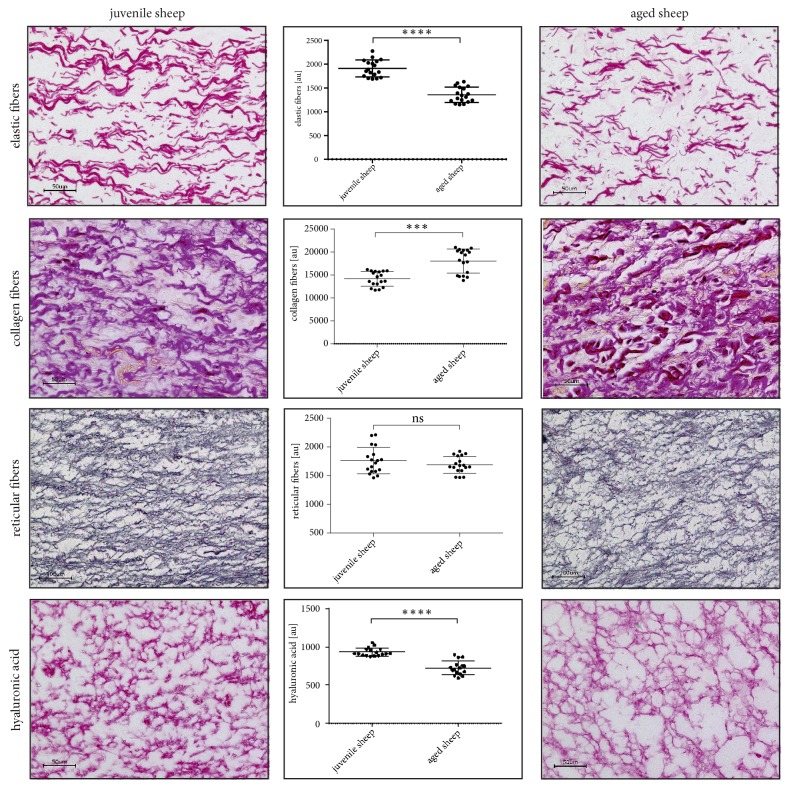
Representative stainings of juvenile and aged sheep vocal fold slides and results of the semiquantitative densitometrical determination of elastic, reticular, and collagen fibers and hyaluronic acid.

Figure 7 shows typical staining of selected connective tissue components in comparison between juvenile and aged larynges. Elastin, which was stained with immunohistochemistry, appeared in red long fibers (juvenile) and visibly shorter fibers in elderly sheep. The amount of elastin appeared lower in aged compared to juvenile larynges. The densitometric analysis of the 6 juvenile and 6 elderly sheep showed a statistically significant (p<0.0001) higher elastin content in the tissue of the juvenile sheep. In contrast, statistically significantly lower collagen levels (p=0.0002) were found in the juvenile sheep VF. In addition, the histological staining of collagen shows slightly shorter and thicker collagen bundles in the aged sheep. Hyaluronic acid was also statistically significantly increased in juvenile sheep (p<0.0001). In the tissue section of the old sheep, smaller gaps in the hyaluronic acid tissue distribution are observable in contrast to the very even distribution in juvenile sheep. No quantitative differences were found in the fine network of reticular fibers; nor did the thickness of the epithelial layer statistical vary between old and juvenile sheep (data not shown). Finally, more fat inclusions being rather randomly scattered was observable in the aged sheep.

In summary, these results show that old sheep exhibit (1) stiffer collagen network due to thicker and shortened fibers; (2) lower tissue viscoelasticity due to reduced HA-level, resulting in reduced water storage and hence in reduced ability to shock absorption due to the constant trauma caused by the vibratory actions of phonation; (3) increased inertance yielding a reduced dynamic range; (4) more random distributed fat inclusion that may to a certain point also explain the reduced systematic behavior in the phonatory experiments; (5) reduced elastin concentration, yielding less changes in vocal fold elongation and hence less overall accurately dynamical measurable differences. These results are consistent with the previously published results in human, rat, and mini-pig aging larynx [24–26].

## 4. Conclusion

Based on the homogenous group of juvenile sheep, we sought to create a comprehensive dataset, describing functional phonatory, micro-CT based anatomical, and histological based parameters. To do so, we applied an elaborated protocol that allowed us to perform extensive examinations in one and the same larynx and to compare the results to a previous study [9].

Juvenile sheep larynges showed much more consistent behaviour than older sheep larynges. In contrast to aged larynges a clear influence of increasing VF pre-stress levels towards applied airflow, f_0_, SPL, and R_B_ was detected (Figure 5): (1) subglottal pressure increased faster when increasing the airflow; (2) the fundamental frequency f_0_ became more sensitive towards changes in subglottal pressure P_S_; (3) SPL became less sensitive towards P_S_; (4) The transglottal flow resistance R_B_ and its sensitivity against P_S_ increased. This means an improved energy transfer from airflow to VF tissue and a change in P_S_-airflow relation [21].

In summary, these preliminary studies showed that (1) the phonatory process in juvenile sheep behaves more consistent towards changes like PS and pre-stress; (2) the phonatory process in juvenile sheep seems to be more effective (i.e., higher R_B_ values); (3) in our methodological conditions, the composition of the vocal folds extracellular matrix varies significantly between sheep age groups, resulting in vocal folds that become less viscoelastic and more rigid in elderly vocal folds. This may be the reason that the dynamic phonatory range in juvenile sheep was higher suggesting a more flexible laryngeal framework.

Next steps will include the investigation of FES treated larynges [7] and their comparison to juvenile and aged larynges.

## Figures and Tables

**Table 1:** Mean and standard deviations separated for larynges and pre-stress levels as well as over all 431 analyzed runs. The phonation threshold pressure (PTP) values are also given.

Larynx / Weight		PTP (Pa)	P_S_ (Pa)	flow (ml/s)	R_B_ (Pa.s/sl)	f_0_ (Hz)	SPL (dB)	CPP_A_ (dB)	CPP_S_ (dB)
L3	w_1_	1032	1931 ± 434	492 ± 274	4816 ± 1788	115 ± 42	72.3 ± 4.8	22.3 ± 5.0	20.7 ± 3.6
	w_2_	1809	3094 ± 698	431 ± 212	8360 ± 2720	88 ± 26	76.4 ± 5.8	20.8 ± 4.7	21.1 ± 4.8
	w_3_	2492	4197 ± 672	492 ± 274	11019 ± 5002	128 ± 33	79.5 ± 4.6	27.7 ± 3.1	26.7 ± 2.0

L4	w_1_	1931	3517 ± 799	492 ± 274	8773 ± 3340	80 ± 16	77.0 ± 4.5	22.5 ± 6.0	22.2 ± 4.9
	w_2_	1979	3758 ± 872	492 ± 274	9348 ± 3488	69 ± 18	77.9 ± 4.9	18.1 ± 2.8	18.6 ± 3.2
	w_3_	1264	3101 ± 975	492 ± 274	7365 ± 2110	63 ± 18	74.7 ± 6.2	17.7 ± 2.7	18.3 ± 2.6

L6	w_1_	1595	3254 ± 966	492 ± 274	7810 ± 2498	90 ± 13	78.5 ± 3.7	29.6 ± 3.2	25.4 ± 5.0
	w_2_	1646	3437 ± 1031	492 ± 274	8214 ± 2529	81 ± 16	77.4 ± 5.9	23.8 ± 6.5	22.7 ± 5.5
	w_3_	1718	3436 ± 958	492 ± 274	8539 ± 2767	83 ± 16	77.7 ± 4.9	24.9 ± 5.8	22.5 ± 5.9

L7	w_1_	610	2446 ± 993	492 ± 274	5329 ± 944	98 ± 33	73.6 ± 6.4	21.0 ± 3.8	19.0 ± 1.9
	w_2_	1701	5728 ± 2173	492 ± 274	13021 ± 3286	149 ± 55	81.0 ± 9.1	26.4 ± 5.2	24.5 ± 4.9
	w_3_	712	3648 ± 1410	492 ± 274	8071 ± 1986	118 ± 40	78.3 ± 11.0	24.8 ± 3.8	23.5 ± 4.5

L8	w_1_	1725	3409 ± 1011	492 ± 274	8178 ± 2596	78 ± 9	73.7 ± 7.8	21.8 ± 4.2	20.5 ± 4.1
	w_2_	1415	3817 ± 1095	492 ± 274	9101 ± 2626	76 ± 21	76.5 ± 7.2	22.9 ± 5.5	20.6 ± 4.3
	w_3_	1496	4103 ± 1422	492 ± 274	9392 ± 2031	69 ± 21	75.5 ± 6.8	23.5 ± 5.7	21.0 ± 4.3

L9	w_1_	1779	4869 ± 1782	492 ± 274	11092 ± 2355	97 ± 22	79.0 ± 8.3	25.2 ± 6.4	24.1 ± 4.8
	w_2_	1364	3859 ± 1131	503 ± 294	9094 ± 2655	85 ± 28	78.4 ± 5.5	19.7 ± 2.2	19.1 ± 1.8
	w_3_	607	3534 ± 1256	435 ± 256	9365 ± 2737	76 ± 19	77.7 ± 7.4	22.8 ± 3.4	20.5 ± 2.8

L10	w_1_	1470	3371 ± 844	492 ± 274	8305 ± 2843	100 ± 18	78.7 ± 5.3	28.8 ± 6.1	26.6 ± 4.8
	w_2_	1722	3746 ± 824	435 ± 256	11384 ± 5379	116 ± 19	80.5 ± 5.5	29.1 ± 5.4	26.0 ± 4.5
	w_ 3_	1913	4216 ± 862	435 ± 256	13143 ± 6755	116 ± 29	81.6 ± 6.8	30.0 ± 4.5	27.0 ± 4.5

L11	w_1_	1477	2791 ± 935	435 ± 256	8021 ± 3602	75 ± 21	74.6 ± 8.2	21.6 ± 3.7	18.3 ± 3.2
	w_2_	1415	3241 ± 802	435 ± 256	9500 ± 3864	93 ± 14	80.4 ± 5.8	28.0 ± 3.8	23.1 ± 4.5
	w_3_	1722	3586 ± 841	435 ± 256	10775 ± 4932	108 ± 26	83.4 ± 5.2	31.1 ± 5.2	28.3 ± 5.2

L12	w_1_	556	1672 ± 605	492 ± 274	3809 ± 836	52 ± 17	68.4 ± 8.6	18.7 ± 2.1	16.7 ± 0.8
	w_2_	1528	3131 ± 641	435 ± 256	9511 ± 4437	67 ± 21	77.8 ± 6.2	23.1 ± 5.3	20.8 ± 4.0
	w_3_	1913	3622 ± 690	435 ± 256	11212 ± 5651	93 ± 24	80.9 ± 4.5	25.5 ± 6.0	23.4 ± 4.2

Overall		*1503 ± 464*	*3502 ± 1290*	*473 ± 261*	*8978 ± 3984*	*91 ± 33*	*77.5 ± 7.1*	*24.1 ± 5.9*	*22.3 ± 5.0*

**Table 2:** TAM volume, percentage difference of the volumes, surface area, percentage difference of the surfaces, surface-area-to-volume ratio of the TAM, and morphometric data of the larynges.

	Larynx no./parameter	L2	L4	L9	L10	L11	L12	Mean ± STD
Volume [mm^3^]	vol_TAM_left	511	647	481	407	583	567	532.7 ± 84.6
	vol_TAM_right	519	671	469	398	593	570	536.8 ± 96.5
	vol_diff [%]	1.65	3.65	2.49	2.33	1.73	0.65	2.08 ± 1.01

Surface [mm^2^]	surf_TAM_left	640	712	578	511	670	747	642.9 ± 87.2
	surf_TAM_right	700	742	589	524	682	745	663.8 ± 88.6
	surf_diff [%]	9.38	4.20	2.01	2.69	1.72	0.21	3.37 ± 3.22

Surface-area-to-volume ratio	TAM left	1.25	1.10	1.20	1.25	1.15	1.32	1.21 ± 0.08
	TAM right	1.35	1.11	1.26	1.32	1.15	1.31	1.25 ± 0.10

Morphometric data [mm]	d1	32.71	32.41	30.06	28.03	32.64	32.27	31.35 ± 1.91
	d2	33.27	33.13	32.15	29.83	34.56	34.03	32.83 ± 1.68
	d3	29.49	32.26	32.4	29.79	33.3	31.26	31.42 ± 1.52
	d4	34.2	35.83	33.86	29.56	38.2	32.24	33.98 ± 2.96
	VFl	18.37	16.77	17.93	16.64	17.72	17.94	17.56 ± 0.70
	VFr	17.48	17.33	18.01	17.02	17.84	17.25	17.49 ± 0.37

## Data Availability

The recorded measured data used to support the findings of this study are available from the corresponding author upon request.
